# Integrating Network Toxicology, Machine Learning, and Molecular Dynamics to Explore the Molecular Network of Triclosan-Induced Acute Myocardial Infarction

**DOI:** 10.3390/ijms27052343

**Published:** 2026-03-02

**Authors:** Qi Zhang, Siwei Zou, Ziyao Yang, Jingbo Cao, Yajuan Fu, Chenjie Feng, Yue Sun, Anning Yang

**Affiliations:** 1 General Hospital of Ningxia Medical University, School of Public Health, Ningxia Medical University, Yinchuan 750004, China; 202304144040@nxmu.edu.cn; 2 NHC Key Laboratory of Metabolic Cardiovascular Diseases Research, Ningxia Medical University, Yinchuan 750004, China; 24132z210112@nxmu.edu.cn (S.Z.); nxmu_5006@163.com (Y.F.); 3 The Second School of Clinical Medicine, Ningxia Medical University, Yinchuan 750004, China; yilin.2004@139.com (Z.Y.); cao.jingbo@outlook.com (J.C.); 4 College of Medical Information and Engineering, Ningxia Medical University, Yinchuan 750004, China; fengchenjie@whu.edu.cn; 5 Ningxia Key Laboratory of Environmental Factors and Chronic Disease Control, Ningxia Medical University, Yinchuan 750004, China

**Keywords:** triclosan, acute myocardial infarction, network toxicology, machine learning, molecular dynamics simulation

## Abstract

Triclosan (TCS) exposure is linked to increased acute myocardial infarction (AMI) risk, but underlying mechanisms remain unclear. Here, we integrated network toxicology, machine learning, molecular simulations, and in vitro assays to delineate this pathway. Differential expression analysis and weighted gene co-expression network analysis (WGCNA) identified 37 candidate genes, which were refined via machine learning to 8 core regulators (including *PTGS2*). Molecular docking and molecular dynamics (MD) simulations confirmed high-affinity, stable binding of TCS to *PTGS2*. In cardiomyocytes, TCS upregulated *PTGS2* and the injury marker *cTnI*, an effect reversed by the *PTGS2* inhibitor celecoxib. These findings establish *PTGS2* as a critical mediator of TCS-induced cardiomyocyte injury, providing a potential therapeutic target for TCS-associated cardiovascular damage.

## 1. Introduction

Triclosan (TCS) is a broad-spectrum antimicrobial compound extensively used in personal care products, medical supplies and food contact materials, resulting in its widespread distribution in the natural environment and chronic human exposure through dermal contact, oral intake and respiratory inhalation [1,2]. Mounting epidemiological evidence has implicated chronic TCS exposure in an elevated risk of cardiovascular disorders, especially acute myocardial infarction (AMI)—a life-threatening condition caused by coronary artery occlusion-induced myocardial necrosis [3,4]. However, the molecular mechanisms governing TCS-induced cardiac toxicity and the subsequent onset of AMI remain largely undefined, which hampers the in-depth elucidation of TCS-associated cardiovascular risks and the development of targeted interventional approaches.

AMI is a typical multifactorial cardiovascular disorder, with its pathogenesis involving aberrant inflammatory cascades, cardiomyocyte injury and thrombotic events that are modulated by complex molecular regulatory networks [5]. Network toxicology and weighted gene co-expression network analysis (WGCNA) have proven to be robust analytical tools for unraveling the molecular underpinnings of environmental toxicant-induced diseases, while machine learning algorithms enable the efficient identification and prioritization of core regulatory genes from complex genomic datasets [6,7]. In addition, molecular docking and molecular dynamics (MD) simulations serve as reliable strategies for predicting and verifying direct binding interactions between exogenous toxicants and their cellular target proteins, and in vitro cellular assays can further provide functional experimental evidence for these predicted molecular mechanisms [8].

Existing studies on TCS-induced cardiovascular toxicity have mostly centered on single-pathway investigations, which fail to characterize the holistic molecular networks driving AMI pathogenesis [9]. Notably, even recent network toxicology or AI-based cardiovascular toxicology studies often stop at candidate target screening—lacking validation of direct toxicant–protein binding or functional verification of the predicted mechanisms [10]. Some AI-driven studies prioritize gene candidates but do not integrate molecular simulations to confirm ligand–receptor interactions, while network toxicology analyses rarely link in silico predictions to in vitro experimental evidence [11]. To address this critical knowledge deficit and bridge the gap in existing integrated approaches, we combined network toxicology, WGCNA, machine learning, molecular simulation techniques and in vitro cellular experiments to comprehensively investigate the mechanistic basis of TCS-induced AMI. We hypothesized that TCS exerts cardiotoxic effects via binding to and modulating key regulatory genes in cardiac-related pathways, thereby perturbing cardiac homeostasis and inducing cardiomyocyte damage that contributes to AMI development.

**Figure 1:**
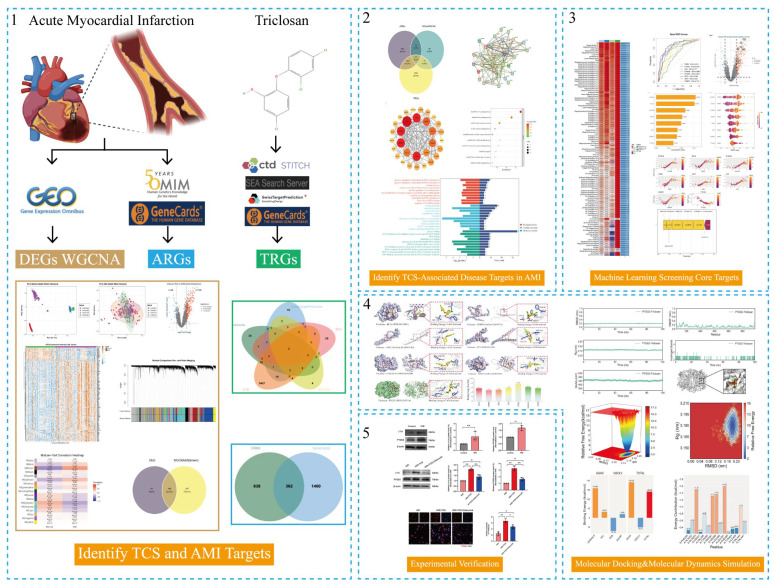
Flowchart of dataset analysis in this paper. Numbers 1–5 represent the key analysis steps: 1 = Identification of TCS and AMI targets; 2 = Identification of TCS-associated disease targets in AMI; 3 = Machine learning screening of core targets; 4 = Molecular docking & molecular dynamics simulation; 5 = Experimental verification. Abbreviations: CTD, Comparative Toxicogenomics Database; STITCH, Search Tool for the Retrieval of Interacting Chemicals; GEO, Gene Expression Omnibus; OMIM, Online Mendelian Inheritance in Man.

In the present study, we initially identified candidate TCS-associated AMI genes through differential expression analysis and WGCNA, then further narrowed down this set to core regulatory genes using machine learning. We subsequently validated the binding specificity and stability between TCS and its top candidate target (*PTGS2*) via molecular docking and MD simulations, and finally verified the functional contribution of *PTGS2* to TCS-induced cardiomyocyte injury using in vitro cellular assays [12]. This study sought to elucidate the underpinning molecular mechanisms of TCS-induced AMI, pinpoint key mediators of TCS-associated cardiac toxicity, and offer a promising therapeutic target for alleviating TCS-induced cardiovascular impairment. The detailed workflow of dataset analysis is illustrated in Figure 1.

## 2. Results

### 2.1. Identification of TCS Target Proteins

**Figure 2:**
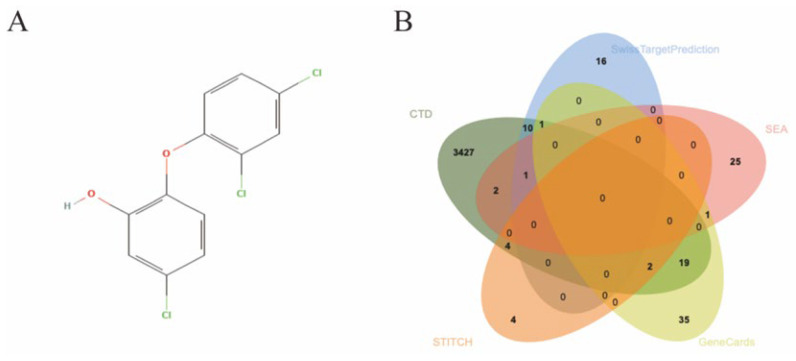
Identification of TCS target proteins. (**A**) Chemical structure of TCS. (**B**) Venn diagram of potential TCS targets predicted by five databases: different colors represent different databases [red = SEA; blue = SwissTargetPrediction; light green = GeneCards; orange = STITCH; dark green = CTD”]; the overlapping area indicates common targets of multiple databases.

The molecular structure of TCS was obtained from the PubChem database (Figure 2A). Potential biological targets of TCS were comprehensively predicted via a combination of the GeneCards, SwissTargetPrediction, SEA, STITCH and CTD databases. Following data integration and de-duplication, a total of 3547 unique potential targets were finally identified (Figure 2B).

### 2.2. Identification of Target Genes Associated with AMI

**Figure 3:**
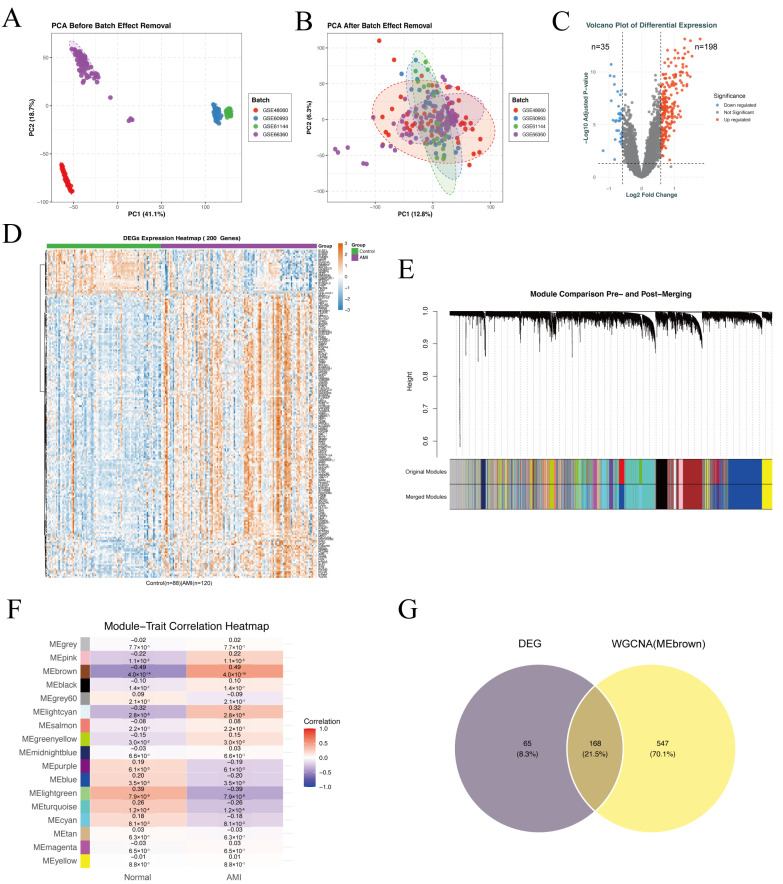
Identification of AMI-related target genes. (**A**) A PCA scatter plot of the GSE48060, GSE60993, GSE61144, and GSE66360 datasets before batch correction, showing clear separation due to batch effects. (**B**) A PCA scatter plot after batch correction demonstrates successful integration of the four datasets and reduction in batch effects. (**C**) A volcano plot displaying differentially expressed genes (DEGs) based on log_2_FC and statistical significance; red dots denote 198 upregulated genes, blue dots 35 downregulated genes, and gray dots non-significant genes. (**D**) A heatmap of DEGs expression patterns across samples, with red indicating upregulation and blue indicating downregulation. (**E**) A gene dendrogram from WGCNA illustrating hierarchical clustering based on co-expression; the color bar below represents distinct gene modules. (**F**) A module–trait relationship heatmap showing correlations between WGCNA-derived modules and the sample trait (Control vs. AMI); each cell contains the correlation coefficient and corresponding *p*-value. (**G**) A Venn diagram intersecting DEGs (purple) and genes from relevant WGCNA modules (yellow), with the overlap representing common genes identified by both methods.

To minimize batch effects, the discovery dataset comprising GSE48060, GSE60993, GSE61144, and GSE66360 was merged, and the gene expression matrix underwent comprehensive normalization. To further validate the effectiveness of batch effect removal, we conducted PCA on gene expression profiles before and after normalization. Prior to normalization, PCA revealed that preprocessed samples clustered distinctly according to their original dataset origins, with non-overlapping groups across different datasets (Figure 3A), indicating substantial batch effects. After batch effect removal and normalization, PCA demonstrated extensive overlap of samples from distinct datasets (Figure 3B), confirming the effective elimination of technical biases. Subsequently, differential expression analysis on the batch-corrected discovery set identified 233 significant differentially expressed genes (DEGs). As visualized in the volcano plot (Figure 3C), 198 DEGs were significantly upregulated (red dots) and 35 were significantly downregulated (blue dots), while gray dots represent genes with no significant expression changes.

The expression patterns of the top 200 most significant DEGs are further displayed via a heatmap (Figure 3D), which clearly shows that DEG expression levels differed markedly between the AMI (*n* = 129) and control (n = 88) groups, with highly consistent expression profiles within each group. For WGCNA, we first systematically evaluated soft threshold exponents 1–20, identifying β = 5 as the minimum value satisfying the scale-free topology criterion (R^2^ ≥ 0.8). Using this threshold, gene clustering was performed based on the topological overlap matrix, and similar modules were partitioned and merged using the dynamic Tree Cut function (parameters: minModuleSize = 30, mergeCutHeight = 0.25). As visualized in Figure 3E (Module Comparison Pre- and Post-Merging), this process ultimately yielded 17 distinct gene co-expression modules, each labeled with a unique color. Furthermore, module-trait association analysis was conducted to identify core modules linked to AMI (Figure 3F). Among all modules, the brown module exhibited the strongest positive correlation with the AMI disease state (correlation coefficient = 1.00, *p* = 1.0 × 10^−100^), indicating its potential key role in AMI pathogenesis. Finally, integrating the 233 DEGs with the 715 genes from the brown core module via Venn diagram analysis yielded 168 overlapping genes, which were defined as the core target genes for AMI (Figure 3G).

### 2.3. Identifying TCS-Related Disease Targets in AMI

**Figure 4:**
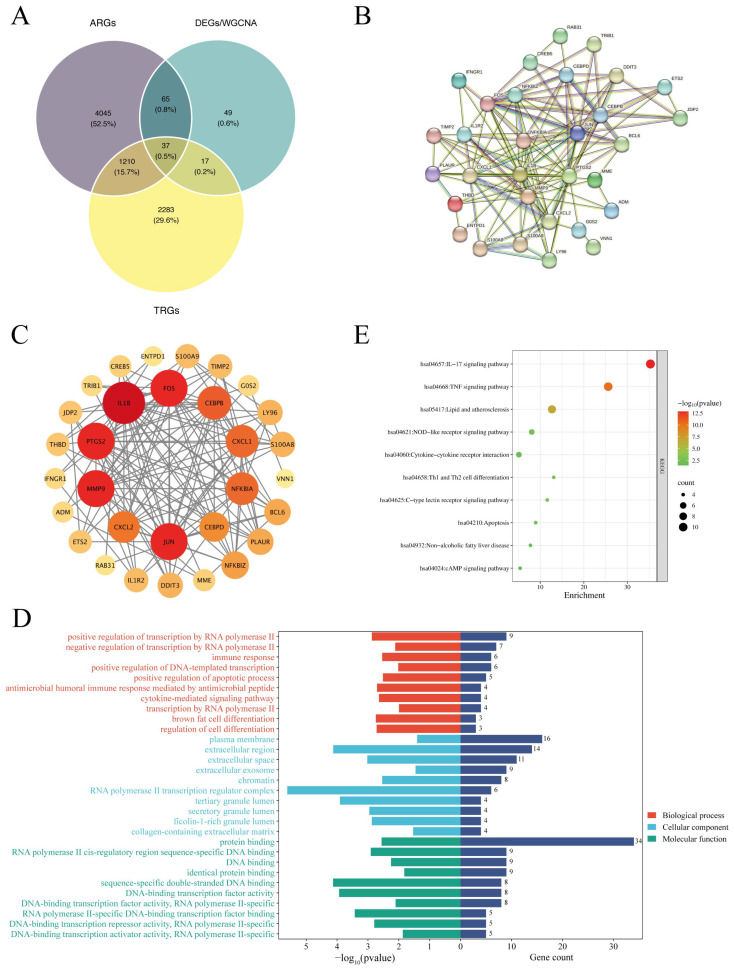
Identification of TCS-associated disease targets in AMI. (**A**) A Venn diagram illustrating the intersection analysis of ARGs (AMI-Related Genes), DEGs/WGCNA core genes, and TRGs (TCS-Related Genes). Different colors indicate distinct analysis modules: blue = DEGs/WGCNA; yellow = TRGs; purple = ARGs. (**B**,**C**) The protein–protein interaction (PPI) network visualizes interactions among overlapping genes. Node size represents the Degree value (larger nodes indicate higher Degree values), colors denote different functional modules, and edges represent interactions between proteins. (**D**) GO enrichment annotates overlapping genes in Biological Process (BP), Cellular Component (CC), and Molecular Function (MF). X-axis = gene count; color gradient = adjusted *p*-value (darker red = higher significance). (**E**) KEGG analysis shows enriched pathways for overlapping genes.

To identify potential key targets mediating the effects of TCS on AMI pathogenesis, we first retrieved AMI-related target genes (ARGs) from the OMIM and GeneCards databases. After merging and removing redundant entries, a total of 5357 AMI-related target genes were identified for subsequent intersection analysis. We then performed a cross-analysis of these ARGs, TCS-related target proteins (TRGs), and differentially expressed genes (DEGs) from our discovery set. As visualized in the Venn diagram (Figure 4A), this intersection analysis identified 37 genes common to all three datasets, which were defined as the core candidate targets involved in TCS-induced AMI. The protein–protein interaction (PPI) network of these 37 core genes was further constructed (Figure 4B), revealing the overall topological structure of their functional interactions and providing a framework for exploring potential regulatory relationships. To further prioritize functionally critical nodes within this network, we identified hub genes by node connectivity (Figure 4C); genes with the highest interaction degrees, including *IL1B*, *FOS*, *JUN*, *PTGS2*, and *MMP9*, were visualized with size proportional to their connectivity, highlighting their potential as key regulatory hubs in TCS-induced AMI. Subsequently, functional annotation of the core genes was performed via GO and KEGG enrichment analyses (Figure 4D–E), providing comprehensive insights into their biological roles. GO enrichment analysis revealed significant enrichment across three categories (Figure 4D): biological processes (BPs) such as positive regulation of transcription by RNA polymerase II, immune response, and cytokine-mediated signaling pathways; cellular components (CCs) including the plasma membrane, extracellular region, and extracellular exosome; and molecular functions (MFs) such as protein binding and DNA binding. KEGG pathway analysis further demonstrated that these core genes were predominantly enriched in inflammation-related pathways (e.g., IL-17 signaling pathway and TNF signaling pathway), lipid metabolism pathways (e.g., lipids and atherosclerosis), and innate immune pathways (e.g., NOD-like receptor signaling pathway and cytokine–cytokine receptor interaction; Figure 4E). Notably, the IL-17 signaling pathway exhibited the highest statistical significance, underscoring the central role of inflammatory dysregulation. Collectively, these results indicate that inflammatory responses and dysregulated lipid metabolism are key biological processes driving TCS-induced AMI pathogenesis.

### 2.4. Identification of Core Genes Involved in TCS-Induced AMI

**Figure 5:**
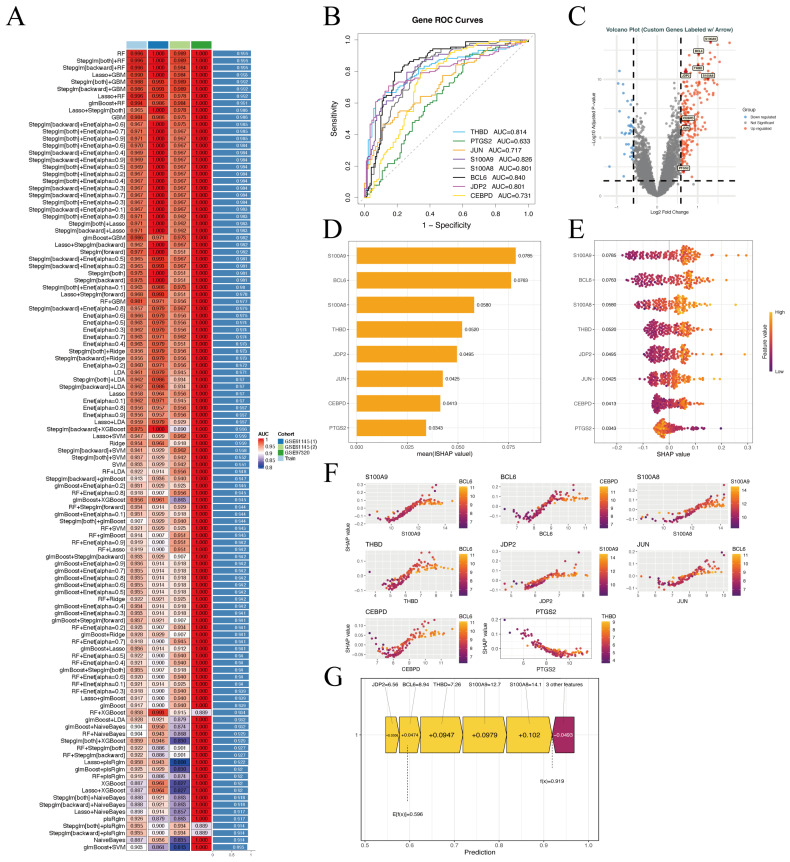
Identification of core genes in TCS-induced AMI. (**A**) Model performance comparison: heatmap of AUC values for different machine-learning models across multiple cohorts. Rows represent models, columns indicate cohorts, and color intensity corresponds to AUC (higher is better). (**B**) ROC curves for eight key candidate genes (*THBD*, *PTGS2*, *JUN*, *S100A9*, *S100A8*, *BCL6*, *JDP2*, *CEBPD*). The x-axis shows the false positive rate, and the y-axis shows sensitivity, different colors distinguish the eight core genes, and the AUC reflects the predictive performance of each gene. (**C**) Volcano plot of DEGs. The x-axis represents log_2_FC, and the y-axis shows −log_10_(*p*-value). Red dots denote significantly upregulated genes, blue dots denote significantly downregulated genes, and grey dots represent non-significant genes; core genes are highlighted with labels. (**D**) Feature importance ranking: a bar plot displays the top genes ranked by their contribution to the model prediction. (**E**) Violin plots illustrating expression distributions of the core genes across experimental conditions. Plot width reflects data density, and colors represent expression levels. (**F**) SHAP value distributions for the core genes, showing their impact on model output in individual samples. (**G**) A SHAP summary plot summarizing the overall directional influence of each gene on the prediction (negative SHAP lowers and positive SHAP raises the predicted outcome).

To identify key genes driving TCS-associated AMI, we performed comprehensive machine learning analysis on 37 candidate targets. Among all models tested, Random Forest (RF) and Stepwise GLM + RF hybrids showed the most robust performance, with AUC and C-index values approaching 1.0 in both training and validation cohorts (Figure 5A). Pruning the RF model yielded 8 core genes: *THBD*, *PTGS2*, *JUN*, *S100A9*, *S100A8*, *BCL6*, *JDP2*, and *CEBPD*. ROC curve analysis confirmed their diagnostic potential (AUC 0.633–0.840), with *BCL6* and *S100A9* performing best (Figure 5B). Volcano plots further showed that these genes were predominantly upregulated in AMI, consistent with their pro-inflammatory roles (Figure 5C). SHAP analysis revealed *S100A9* and *BCL6* as the most influential predictors (Figure 5D), while *CEBPD* and *JDP2* exhibited bidirectional effects (Figure 5E). Interaction analysis uncovered nonlinear relationships: *S100A8* and *S100A9* acted synergistically to increase risk, *THBD* peaked in predictive value at low expression, and *BCL6* showed a bimodal effect (Figure 5F). Finally, SHAP force plots visualized how *S100A8*, *THBD*, and *BCL6* drove elevated risk in a representative sample, with *JDP2* as the sole negative regulator (Figure 5G). Collectively, these findings validate our machine learning models, identify diagnostically relevant core genes, and reveal their complex regulatory interactions, providing biomarkers and mechanistic insights into TCS-associated AMI.

### 2.5. Molecular Docking Validation of TCS–Core Gene Interactions

**Figure 6:**
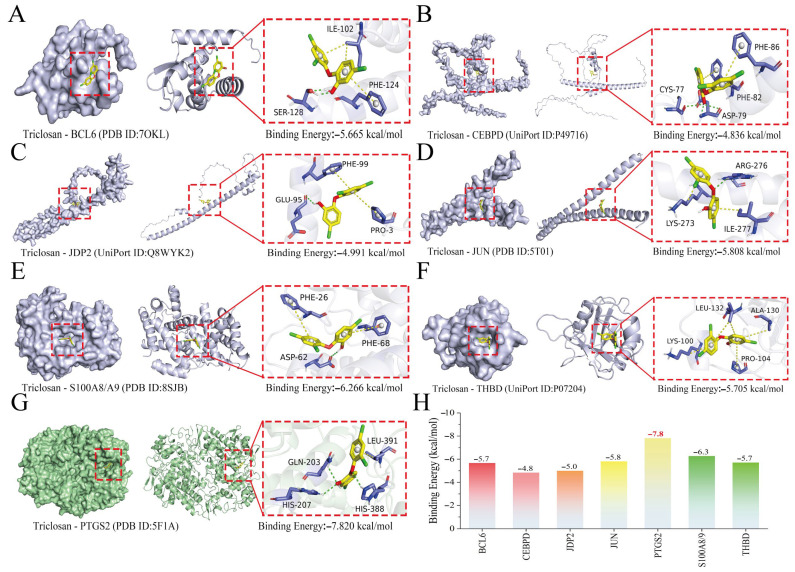
Molecular docking validation of TCS–core gene interactions. (**A**–**G**) Molecular docking poses and predicted binding affinities between TCS and core gene products: (**A**) *BCL6*, (**B**) *CEBPD*, (**C**) *JDP2*, (**D**) *JUN*, (**E**) *S100A8*/A9 heterocomplex, (**F**) *THBD*, and (**G**) *PTGS2*. The core protein is shown in purple, with key binding regions highlighted in orange-red. Yellow dashed lines represent the hydrogen bonds formed between the ligand and the amino acid residues of the protein, and the adjacent numbers indicate the corresponding bond lengths, which reflect the strength of the interaction. The ligand is illustrated in blue to highlight its three-dimensional chemical structural characteristics. (**H**) Summary of calculated binding energies for all TCS–core gene complexes.

To validate potential binding interactions between TCS and the core gene-encoded proteins (*THBD*, *PTGS2*, *JUN*, *S100A9/A8*, *BCL6*, *JDP2*, and *CEBPD*) identified in our prior analyses, we performed in silico comprehensive molecular docking simulations (Figure 6A–F). Binding energies < 0 kcal·mol^−1^ are indicative of spontaneous ligand–receptor association, which occurs without requiring external energy input. Values ≤ −5 kcal·mol^−1^ denote excellent binding affinity, while those ≤ −7.2 kcal·mol^−1^ indicate high-affinity interactions with strong functional relevance. Our docking simulations confirmed that TCS exhibited excellent binding affinity (ΔG ≤ −5 kcal·mol^−1^) to seven of the eight target proteins, including *THBD*, *PTGS2*, *JUN*, *S100A9/A8*, *BCL6*, and *JDP2* (Figure 6A,C–F). Notably, TCS demonstrated the highest binding affinity for *PTGS2*, with a calculated binding energy of −7.820 kcal·mol^−1^ (ΔG ≤ −7.2 kcal·mol^−1^), confirming a high-affinity interaction (Figure 6G). Structural visualization further revealed that TCS formed three hydrogen bonds with *PTGS2* residues GLN-203, HIS-207, and HIS-388 (green dashed lines) and a hydrophobic interaction with LEU-391 (yellow dashed line). This high-affinity binding suggests that TCS may directly modulate the structural conformation and biological activity of *PTGS2*, a key enzyme driving pro-inflammatory prostaglandin synthesis in AMI pathogenesis. Detailed docking parameters, including PDB/Uniprot IDs, binding energies, and key interacting residues, are summarized in Table 1 and Figure 6H.

### 2.6. Molecular Dynamics Simulation

**Figure 7:**
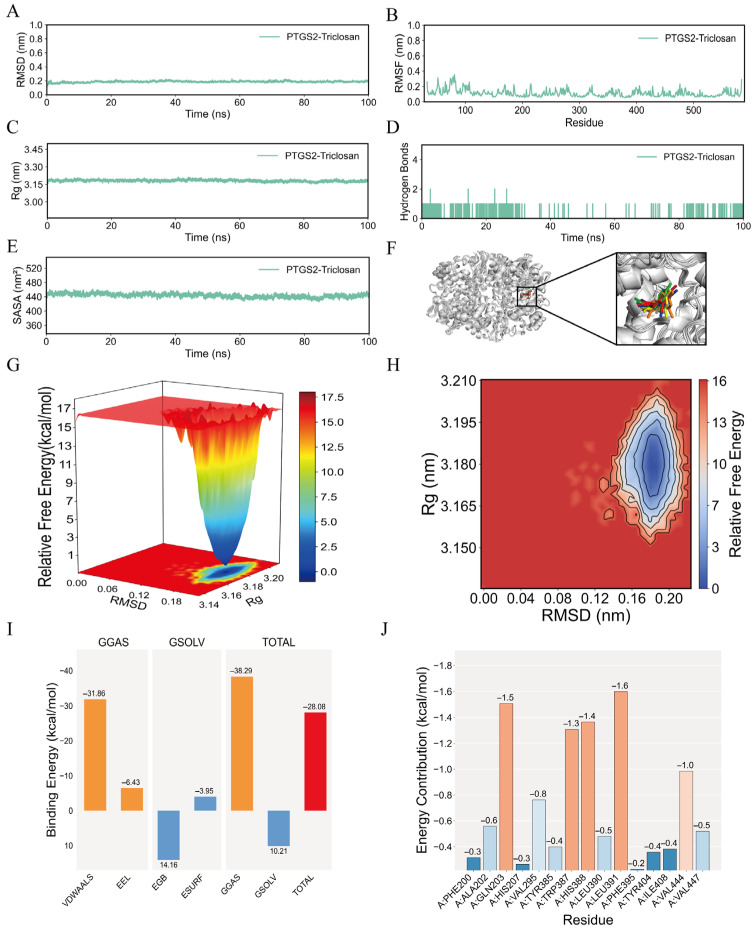
Molecular dynamics simulation of *PTGS2* and TCS complexes. (**A**) RMSD curve of the *PTGS2* protein-TCS complex. (**B**) RMSF curve of the *PTGS2* protein-TCS complex. (**C**) Rg curve of the *PTGS2* protein-TCS complex. (**D**) Fluctuation curve of hydrogen bond formation between *PTGS2* protein and TCS. (**E**) SASA curve of the *PTGS2*-TCS complex. (**F**) Structural comparison of the *PTGS2*-TCS complex at five time points (0, 25, 50, 75, 100 ns) during molecular dynamics simulation. The red, green, blue, yellow, and orange small molecules correspond to the TCS molecular structures at 0, 25, 50, 75, and 100 ns, respectively. (**G**,**H**) Free energy distribution plots of the *PTGS2*-TCS complex. Different colors are used to distinguish distribution contours and data density. (**I**) Average binding free energy of *PTGS2* with TCS. VDWAALS, EEL, EGB, ESURF, GGAS, GSOLV, and TOTAL represent van der Waals forces, electrostatic energy, polar solvation energy, nonpolar solvation energy, molecular mechanics term, solvation energy term, and average binding free energy, respectively. Different colors represent different energy items for clear distinction. (**J**) Energy contribution of amino acid residues in the *PTGS2* protein involved in TCS binding.

The root means square deviation (RMSD) profile demonstrated that fluctuations in the *PTGS2*-TCS complex converged to a stable plateau at approximately 0.2 nm, indicating minimal structural deviations and confirming the overall conformational stability of the complex (Figure 7A). This stable binding may enable sustained occupancy of *PTGS2*’s active site in cardiovascular cells, potentially disrupting prostaglandin homeostasis—a critical regulator of vascular function [13]. Root mean square fluctuation (RMSF) analysis revealed that residue-level fluctuations remained within 1 nm, indicating minimal conformational perturbations and a negligible impact of TCS on the global stability of *PTGS2* (Figure 7B). The radius of gyration (Rg) profile remained stable at approximately 3.20 nm throughout the simulation, reflecting a compact and conformationally stable tertiary structure of the *PTGS2*-TCS complex (Figure 7C). The number of persistent hydrogen bonds between TCS and *PTGS2* remained consistently at ~1 throughout the simulation, which may reinforce complex stability and enable sustained modulation of *PTGS2*-mediated pro-inflammatory signaling—a key driver of vascular inflammation and atherosclerosis (Figure 7D). The solvent-accessible surface area (SASA) profile exhibited stable fluctuations within the 450 nm^2^ range, confirming the inherent structural stability of the *PTGS2*-TCS complex (Figure 7E). Visualization of the binding pose at five time points (0–100 ns) revealed no significant positional shifts, further validating the high stability of the *PTGS2*-TCS complex (Figure 7F). The free energy landscape (FEL) exhibited a single, well-defined energy minimum, indicating a thermodynamically stable conformation of the complex (Figure 7G,H). The average binding free energy was calculated to be −28.08 kcal·mol^−1^, confirming a high-affinity interaction between TCS and *PTGS2* (Figure 7I). This robust binding affinity suggests that TCS may target *PTGS2* even at environmentally relevant concentrations previously detected in human biological fluids [13]. Per-residue binding energy analysis identified key interacting residues (LEU-391, GLN-203, HIS-388, and TRP-387) with favorable binding energies ranging from −1.6 to −1.3 kcal·mol^−1^ (Figure 7J). These interactions may alter the conformation of *PTGS2*’s active site, potentially shifting prostaglandin synthesis toward pro-inflammatory isoforms while suppressing vasoprotective ones. Notably, these key binding residues were consistent with our prior molecular docking results, with no significant positional shifts observed, further reinforcing the stability of the *PTGS2*-TCS complex. This stable active-site conformation may facilitate the sustained enzymatic activity of *PTGS2* in the pathological progression of AMI.

### 2.7. Experimental Validation of PTGS2 as a Mediator of TCS-Exacerbated Cardiomyocyte Injury

**Figure 8:**
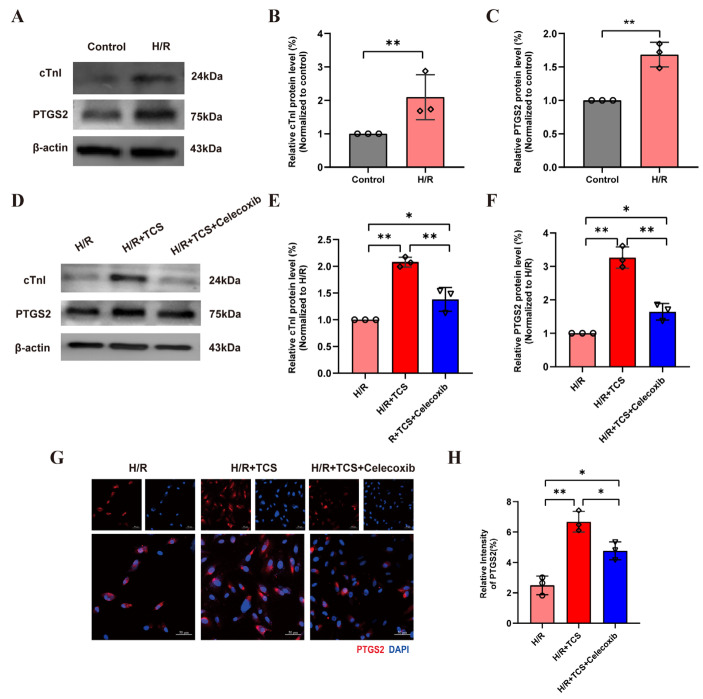
*PTGS2* mediates TCS-exacerbated cardiomyocyte injury. (**A**) Western Blot and (**B**,**C**) semiquantitative analysis of *PTGS2* and *cTnI* in H9C2 cells treated with hypoxia-reoxygenation (H/R). (**D**) Western Blot and (**E**,**F**) semiquantitative analysis of *PTGS2* and *cTnI* in H9C2 cells subjected to H/R, pre-treated with 5 μmol/L celecoxib for 1 h, and then treated with 10 μmol/L TCS for 24 h. (**G**) Immunofluorescence staining of *PTGS2* (red) and DAPI (nuclei, blue) in the three groups (scale bar = 50 μm). (**H**) Quantitative analysis of *PTGS2* mean fluorescence intensity. * *p* < 0.05, ** *p* < 0.01 (statistical significance); n = 3 (number of independent experimental replicates).

To confirm the functional relevance of *PTGS2* in TCS-mediated cardiomyocyte in-jury, we established an AMI cell model by subjecting H9c2 cardiomyocytes to H/R, then assessed cellular injury and *PTGS2* expression. Western Blot results showed that H/R treatment significantly elevated protein levels of the cardiomyocyte injury marker *cTnI* and *PTGS2* in H9c2 cells (Figure 8A–C). These data confirmed successful establishment of the H/R-induced cardiomyocyte injury model, with concurrent upregulation of *PTGS2*. Next, we evaluated the effect of TCS on H/R-treated cells and the result showed that treatment with 10 μmol/L TCS (H/R + TCS group) further increased *cTnI* and *PTGS2* protein levels. This indicates that TCS exacerbates H/R-induced cardiomyocyte injury and promotes *PTGS2* upregulation. To verify the role of *PTGS2*, we pre-treated cells with the selective *PTGS2* inhibitor celecoxib (5 μmol/L) before TCS exposure. Relative to the H/R+TCS group, celecoxib treatment (H/R + TCS + Celecoxib group) significantly reduced both *cTnI* and *PTGS2* protein levels (Figure 8D–F), confirming that *PTGS2* inhibition mitigates TCS-exacerbated cardiomyocyte damage. Immunofluorescence staining further validated that *PTGS2* was markedly elevated in the H/R + TCS group versus the H/R group, while celecoxib treatment reduced *PTGS2* protein level (Figure 8G,H). Collectively, these results demonstrate that *PTGS2* is a critical mediator of TCS-induced cardiomyocyte injury.

## 3. Discussion

Triclosan (TCS) is a widely used phenolic antimicrobial agent, with its ubiquitous environmental presence and chronic human exposure raising concerns about potential cardiovascular toxicity [14,15]. Epidemiological and toxicological studies have linked TCS exposure to an increased risk of acute myocardial infarction (AMI), a life-threatening cardiovascular disorder driven by complex pathological cascades involving inflammation, cardiomyocyte injury, and thrombosis [16,17]. However, the specific molecular mechanisms connecting TCS exposure to AMI pathogenesis remain poorly defined, creating a critical knowledge gap in environmental toxicology and cardiovascular research. To address this gap, we employed an integrated strategy combining network toxicology, weighted gene co-expression network analysis (WGCNA), machine learning, molecular simulations, and in vitro cellular assays to systematically dissect the mechanistic basis of TCS-induced AMI.

Our study first identified 37 candidate genes associated with both TCS exposure and AMI via network toxicology and WGCNA, an approach proven effective for unraveling complex molecular networks in toxicant-induced diseases [18,19]. We then applied combinations of machine learning algorithms to refine these candidates into eight core regulatory genes (*S100A9*, *BCL6*, *S100A8*, *THBD*, *JDP2*, *JUN*, *CEBPD*, and *PTGS2*), which exhibited robust diagnostic value for AMI in independent datasets. The combined diagnostic model achieved an ideal AUC, with *BCL6*, *S100A9*, and *THBD* showing the strongest single-gene performance, highlighting the potential of these core genes as novel diagnostic biomarkers for TCS-associated AMI. Functional enrichment analysis further confirmed that these genes are enriched in key AMI-relevant pathways, including inflammatory responses, lipid metabolism, and atherosclerosis—consistent with the well-documented role of inflammation and metabolic dysregulation in AMI progression [20,21]. This finding suggests that TCS may exert its cardiotoxic effects by disrupting these critical biological pathways through the modulation of core regulatory genes.

Among the eight core genes, *PTGS2* (*cyclooxygenase-2*, *COX-2*)—a key enzyme in prostaglandin synthesis—emerged as the most functionally relevant target of TCS. Molecular docking analysis revealed a high-affinity interaction between TCS and *PTGS2*, with a binding energy of −7.820 kcal·mol^−1^, indicating a stable ligand–receptor association. This observation was further validated by molecular dynamics (MD) simulations, which demonstrated that the TCS-*PTGS2* complex maintains structural stability throughout the simulation period: RMSD values fluctuated stably within 0.2 nm, RMSF values remained below 1 nm, and the average binding free energy was calculated to be −28.08 kcal·mol^−1^. This robust binding stability and conserved active-site conformation support the sustained modulation of *PTGS2* by TCS in cardiac and vascular cells. Key binding residues (LEU-391, GLN-203, HIS-388, TRP-387) were consistent between docking and MD simulations, confirming the specificity of this interaction. These specific residue interactions may alter the catalytic microenvironment of *PTGS2*, which is critical for its enzymatic regulation of prostaglandin synthesis in AMI. These in silico findings are particularly meaningful, as *PTGS2* is well known to play a central role in AMI pathogenesis by regulating prostaglandin balance—its upregulation in atherosclerotic plaques disrupts the ratio of pro-thrombotic thromboxane A2 and vasoprotective prostaglandin I2, promoting plaque instability and thrombogenesis [22]. Our results suggest that TCS may directly bind to *PTGS2*, alter its enzymatic activity, and disrupt prostaglandin homeostasis, thus amplifying the pro-inflammatory and pro-thrombotic pathological cascades in AMI, thereby triggering the pathological cascades leading to AMI.

To confirm the functional relevance of the TCS-*PTGS2* interaction, we performed in vitro cellular assays using H9c2 cardiomyocytes. Consistent with our in silico predictions, TCS exposure significantly upregulated *PTGS2* expression, accompanied by increased levels of the cardiac injury marker *cTnI*, indicating that TCS induces cardiomyocyte injury. Importantly, treatment with celecoxib, a selective *PTGS2* inhibitor, not only suppressed TCS-induced *PTGS2* upregulation but also attenuated *cTnI* expression, directly confirming that *PTGS2* mediates TCS-induced cardiomyocyte injury. This finding provides critical experimental evidence linking TCS exposure to cardiac toxicity via *PTGS2* modulation, filling the mechanistic gap between TCS environmental exposure and AMI development.

The integrated approach employed in this study offers several advantages over previous research on TCS-induced cardiovascular toxicity, which has primarily focused on single-pathway or single-molecule analyses. By combining multi-omics target screening, machine learning, and experimental validation, we were able to identify a set of core regulatory genes and pinpoint *PTGS2* as the key functional target, providing a comprehensive molecular framework for TCS-induced AMI. This framework suggests that TCS may induce AMI through a multi-gene, multi-pathway mechanism: direct binding to *PTGS2* to disrupt prostaglandin synthesis, and indirect modulation of other core genes (e.g., *S100A8*/A9, *THBD*) to amplify inflammation and disrupt vascular homeostasis. This holistic understanding of TCS cardiotoxicity is a significant advancement in the field, as it highlights the complexity of environmental toxicant-induced diseases and the need for integrated approaches to unravel their mechanisms.

One notable limitation of the present study is its proteocentric focus, which prioritizes the interaction between TCS and target proteins while overlooking TCS’s non-specific membranotropic effects—a critical cytotoxic pathway. TCS is known to intercalate into lipid bilayers, disrupting biological membrane integrity and permeability [23,24]. This mechanism may independently induce cytotoxicity or synergize with the *PTGS2*-mediated pathway to exacerbate TCS-induced cardiotoxicity and AMI progression. Beyond this mechanistic oversight, several additional limitations should be acknowledged. First, the transcriptomic data used for target screening were derived from public databases with a limited sample size and homogeneous population characteristics, which may limit the generalizability of our findings to diverse ethnicities and age groups. Second, traditional cardiovascular risk factors (e.g., smoking, hypertension, diabetes) were not fully controlled for in the analysis, leaving open the possibility that these confounding factors may influence the expression of core genes and their association with AMI risk. Third, the present study lacks in vivo animal experiments to validate the identified mechanisms in a physiological context; future studies should employ TCS-exposed animal models of AMI to confirm the in vivo relevance of the TCS-*PTGS2* interaction. Finally, clinical cohort studies are needed to verify the association between TCS exposure levels, core gene expression, and AMI risk in human populations, which will further strengthen the translational value of our findings.

## 4. Materials and Methods

### 4.1. Acquisition of Disease-Related Targets

The NCBI GEO database was searched using the keyword “Acute Myocardial Infarction,” prioritizing human samples to ensure clinical relevance to the study’s research question. Six datasets (GSE48060, GSE60993, GSE61144, GSE66360, GSE97320, GSE61145) were selected: GSE48060, GSE60993, GSE61144, and GSE66360 served as discovery cohorts, while GSE97320 and GSE61145 were used as validation cohorts to verify the stability and reliability of core genes.

Data processing was performed using R software 4.3.1 with core packages including sva 3.50.0 and limma 3.58.1. Preprocessed gene expression matrices (txt format) were initially normalized via the normalize Between Arrays function from the limma package 3.58.1. For datasets where the 99th percentile of expression values exceeded 100, or the difference between maximum and minimum values exceeded 50 with the 25th percentile > 0, log_2_ transformation (log_2_(x + 1)) was applied to mitigate skewed distribution effects. Batch effects were eliminated using the ComBat function from the sva package when merging multiple datasets. Principal Component Analysis (PCA) was conducted to validate correction efficacy using the factoextra package 1.0.7: PCA plot before batch correction, and the plot post-correction. Clustering trends of AMI and control group samples were compared pre- and post-correction to confirm a significant reduction in batch effects.

Concurrently, AMI-related targets were collected from two authoritative databases to ensure comprehensiveness: the Online Mendelian Inheritance in Man (OMIM) database, which contains selective genes with well-established clinical and genetic associations with human diseases; and the GeneCards database, which integrates multi-source gene–disease associations and assigns a “Relevance Score” to quantify association strength. For GeneCards, the top 2000 genes with the highest Relevance Scores were selected to prioritize robust AMI-linked targets and exclude weakly associated genes that might introduce noise. Duplicate targets from the two databases were removed via Venn diagram integration, yielding a final set of high-confidence disease-related targets for subsequent analyses.

### 4.2. Acquisition of Chemical Components and Targets of TCS

TCS was characterized by integrating multi-database data: its physicochemical properties and biological parameters were retrieved from PubMed, and its standardized two-dimensional structure (SMILES:C1=CC(=C(C=C1Cl)O)OC2=C(C=C(C=C2)Cl)Cl) was retrieved from the PubChem database.

To identify TCS-associated biological targets, “Triclosan” was used as the keyword in five complementary databases, with explicit selection criteria for each to ensure target reliability: GeneCards database (Relevance Score ≥ 0.5, signifying meaningful TCS association); SwissTargetPrediction database (probability > 0, prioritizing high-confidence predictions based on structural similarity and ligand-binding profiles); Similarity Ensemble Approach (SEA) database (Fit Score ≥ 0.8, leveraging chemical similarity to known ligands for target prediction); STITCH database (confidence score ≥ 0.7, integrating experimental evidence and computational predictions of chemical–protein interactions); Comparative Toxicogenomics Database (CTD, targets annotated with “direct interaction” with TCS, excluding indirect/inferred associations to focus on direct molecular targets). Duplicate targets from the five databases were integrated and removed, generating a high-quality set of TCS-related targets for subsequent intersection analysis with AMI-related targets.

### 4.3. Differential Gene Expression Analysis

Based on the corrected gene expression matrix, differential expression analysis between AMI and control groups was performed using the limma package 3.58.1: a design matrix and comparison matrix were constructed, with screening thresholds set at adjusted *p*-value (FDR) < 0.05 and |log_2_ fold change (log_2_FC)| > 0.585 (corresponding to a 1.5-fold expression change). Volcano plots were generated using the ggplot2 package 3.4.4 (x-axis: log_2_FC; y-axis: −log_10_(FDR)), with distinct colors indicating “significantly upregulated genes” (red), “significantly downregulated genes” (blue), and “non-significant genes” (gray). Heatmaps of differentially expressed genes (DEGs) were generated using the heatmap package 1.0.12: the top 50 most significantly DEGs were selected, clustered by sample group, to visually display expression pattern differences between the two groups.

### 4.4. Weighted Gene Co-Expression Network Analysis

Gene co-expression networks were constructed using the WGCNA package 1.72-1 to identify core modules and hub genes associated with AMI.

Sample quality control: Sample clustering trees were computed based on gene expression matrices, and outlier samples were removed via the cutreeStatic function (height threshold = 0.25) to ensure clustering stability of remaining samples.

Soft threshold determination: Powers ranging from 1 to 20 were tested; the minimum power (e.g., power = 6) was selected using the pick Soft Threshold function to meet the “scale-free network topology (R^2^ ≥ 0.8)” criterion, ensuring alignment with biological network characteristics.

Module partitioning: Hierarchical clustering was performed based on the Topological Overlap Matrix (TOM), and co-expression modules were partitioned using the dynamic Tree Cut function (parameters: min Module Size = 30, merge Cut Height = 0.25). Similar modules were merged, and each module was labeled with a distinct color.

Module–Trait Association Analysis: Module Eigenvalues (MEs) were calculated for each module. Pearson correlation analysis was used to evaluate ME correlation with “AMI disease state” (case group = 1, control group = 0). Modules with |r| > 0.5 and *p* < 0.05 were selected as “AMI-related core modules.”

Hub Gene Selection: Module membership connectivity (kME) was calculated for each gene within core modules. Genes with kME > 0.8 were designated as module hub genes, recognized as key regulators of module function.

### 4.5. Identification of TCS-Associated Disease Targets

Cross-analysis of DEGs/WGCNA core genes, AMI-related genes (ARGs), and TCS-related genes (TRGs) identified core TCS-AMI targets, which were visualized via Venn diagrams.

### 4.6. Construction of Protein Interaction Networks and Identification of Core Targets

The intersection targets were submitted to the STRING database (https://string-db.org/, accessed on 15 July 2025), with parameters set to “Multiple proteins” and “Organism = Homo sapiens” to construct the protein–protein interaction (PPI) network. The TSV-formatted file was imported into Cytoscape 3.10.1 software. Network topology analysis was performed using the Network Analyzer plugin 4.4.0 to obtain Degree values. Targets were sorted by Degree values, and network modules were visualized.

### 4.7. Functional Enrichment Analysis

The intersecting targets were uploaded to the DAVID database (https://davidbioinformatics.nih.gov, accessed on 15 July 2025), with “Functional Annotation” selected and species set to “Homo sapiens” for GO functional analysis and KEGG pathway analysis. Results were sorted by ascending *p*-value. The top 20 results for Biological Process (BP), Molecular Function (MF), and Cellular Component (CC) (GO analysis) and the top 10 atherosclerosis-related KEGG pathways were visualized using the MicroBioinformatics Network Platform.

### 4.8. Machine Learning-Based Core Gene Screening

A multi-algorithm machine learning framework was established to systematically screen core genes associated with TCS-related AMI. Leveraging gene expression profiles from the training cohort, 113 predictive models were developed using ten conventional machine learning techniques: Lasso, Support Vector Machine (SVM), Random Forest (RF), glmBoost, Stepglm, Ridge, Elastic Net (Enet), Gradient Boosting Machine (GBM), Linear Discriminant Analysis (LDA), XGBoost, and Naive Bayes. Hyperparameter tuning was performed via 5-fold cross-validation with stratified sampling to maintain balanced class distribution between training and internal validation sets. Model efficacy was evaluated using key metrics including area under the ROC curve (AUC), accuracy, and F1-score. Top-performing models were integrated using a stacking-based ensemble strategy. Models with high predictive confidence (AUC > 0.9) were retained, and feature genes contributing to these models were ranked by occurrence frequency to shortlist candidate core genes. Expression profiles of the resulting gene set were visualized using the pheatmap package 1.0.12.

### 4.9. Molecular Docking Analysis

To further validate the robustness of the finding, molecular docking analysis was performed between TCS and core targets. The 3D structure of each protein was retrieved from the PDB database and inspected using PyMOL 2.3.0 software. The 3D structure of the TCS ligand was downloaded from the PubChem database and refined using the MMFF94 force field in OpenBabel 3.1.1 software to obtain the optimal low-energy molecular structure. Proteins were hydrogenated using AutoDock Tools 1.5.6, while small molecules were hydrogenated and rotatable bonds were defined; all files were saved in pdbqt format. Molecular docking parameters were configured using the Grid module, with semi-flexible docking selected as the method. The exhaustiveness level was set to 25, and the Lamarckian genetic algorithm was employed. AutoDock Vina 1.2.5 was used to perform molecular docking, yielding binding free energy values and docking result files. Notably, the semi-flexible docking approach does not account for protein structural flexibility, environmental temperature, pressure, or solvent effects. To further validate binding affinity and stability between TCS and core targets, a 100 ns molecular dynamics simulation was conducted on the TCS–core target complex.

### 4.10. Establishment and Analysis of Molecular Dynamics Simulation Models

Molecular dynamics simulations of the *PTGS2*-TCS complex were conducted using Gromacs 2024.4 software. The Amber14sb force field was used for proteins, Gaff2 for ligands, and the TIP4P water model was employed to solvate the protein–ligand system. A periodic water box with a 1.2 nm buffer was constructed. The Particle Mesh Electrostatic (PME) method was used to calculate long-range electrostatic interactions. A Monte Carlo ion placement method was used to introduce an appropriate number of sodium and chloride ions to neutralize the system charge. Prior to the main simulation, three-step system energy minimization and equilibration were performed: (1) energy minimization for each system using 50,000 steps of the steepest descent algorithm (stopped when maximum force < 1000 kJ/mol/nm); (2) pre-equilibration for 50,000 steps at 2 fs time step under constant particle count, volume, and temperature (310 K); (3) pre-equilibration for 50,000 steps at 2 fs time step under constant particle count, pressure (1 bar), and temperature (310 K) for the entire system. Following minimization and equilibration, a 100 ns molecular dynamics simulation was conducted at 2 fs time step without constraints, with structural coordinates saved every 10 ps.

Trajectories of the *PTGS2*-TCS complex were analyzed to evaluate root mean square deviation (RMSD), root mean square fluctuation (RMSF), radius of gyration (Rg), solvent-accessible surface area (SASA), and the number of hydrogen bonds between proteins and ligands. Relative free energy distribution and complex structures at 0, 25, 50, 75, and 100 ns were also examined. The MM/GBSA method was used to calculate the average binding free energy between proteins and ligands.

### 4.11. Cell Experiment Validation

To confirm the regulatory role of *PTGS2* in TCS-induced AMI, H9C2 *rat cardiomyocytes* cardiomyocytes were used to construct a cell injury model mimicking AMI. The effects of TCS and a *PTGS2* inhibitor were evaluated via immunofluorescence analysis and Western Blot.

#### 4.11.1. Cell Culture and AMI Model Construction

H9C2 cells were maintained in DMEM high-glucose medium supplemented with 10% fetal bovine serum and 1% penicillin–streptomycin at 37 °C with 5% CO_2_. Cells in the logarithmic growth phase were seeded in 6-well plates at a density of 7 × 10^3^ cells/well. When cell confluence reached 60%, the AMI model was constructed via hypoxia-reoxygenation (H/R): cells were washed twice with PBS, incubated in serum-free medium, sealed in a hypoxic culture chamber for 16 h, then transferred to normal culture conditions with complete medium for 2 h of reoxygenation.

#### 4.11.2. Cell Grouping and Treatment Methods

H9C2 cells were divided into four groups: (1) Control group (normal H9C2 cells without hypoxia–reoxygenation or other treatments); (2) H/R group (cells treated with hypoxia–reoxygenation); (3) H/R + TCS group (cells treated with 10 μmol/L TCS for 24 h after hypoxia–reoxygenation [3,25]); (4) H/R + Celecoxib + TCS group (*PTGS2* inhibitor: Celecoxib[Viatris Pharmaceuticals LLC, Vega Baja, Puerto Rico, USA]): cells were subjected to hypoxia–reoxygenation (H/R) first, then pre-treated with 5 μmol/L celecoxib for 1 h, followed by 10 μmol/L TCS treatment for 24 h.

#### 4.11.3. Immunofluorescence Analysis of *PTGS2* Protein Expression

After intervention, cells were fixed with 4% paraformaldehyde for 30 min, washed with PBS, blocked with 5% bovine serum albumin (BSA) for 1 h, and incubated with PTGS2 primary antibody (1∶100 dilution) overnight at 4 °C. Cells were then incubated with Alexa Fluor 488-labeled secondary antibody (1∶200 dilution) at 37 °C for 1 h in the dark. Nuclei were stained with DAPI, and images were captured via laser confocal microscopy. Quantitative analysis was performed using ImageJ software 1.54f: 5 random fields per image were selected to measure the mean fluorescence intensity (MFI) of *PTGS2*, with the Control group set as the reference (100%).

#### 4.11.4. Western Blot Detection of *PTGS2* and *cTnI* Protein Expression

Total cellular protein was extracted, and protein concentrations were quantified using a BCA assay kit (Thermo Fisher Scientific, Waltham, MA, USA). Protein samples (10 μg) were separated by SDS-PAGE, transferred to PVDF membranes, blocked with 5% skimmed milk for 2 h, and incubated with PTGS2 (1∶1000), cTnI (1∶1000), and GAPDH (1∶5000) primary antibodies overnight at 4 °C. After washing, membranes were incubated with HRP-labeled secondary antibody (1∶2000) at room temperature for 2 h, visualized using an ECL detection system, and grayscale analysis was performed using Image Lab software 6.1.

### 4.12. Statistical Analysis

All statistical analyses were performed using R software (version 4.3.1) and GraphPad Prism 10.0. Quantitative data are presented as the mean ± standard deviation, with intergroup comparisons conducted using independent sample *t*-tests (two groups) or one-way analysis of variance (ANOVA, multiple groups). Categorical data are expressed as a number (percentage) and compared via chi-square tests. Pearson’s correlation coefficient was used for correlation analysis. All statistical tests were two-tailed, and a *p* value < 0.05 was considered statistically significant.

## 5. Conclusions

The present study systematically dissects the molecular mechanisms of TCS-induced AMI using an integrated multi-disciplinary approach. We identified eight core regulatory genes associated with TCS exposure and AMI, and validated *PTGS2* as the key functional target mediating TCS-induced cardiomyocyte injury. Our findings not only advance the current understanding of TCS-induced cardiovascular toxicity but also provide novel diagnostic biomarkers and a potential therapeutic target (*PTGS2*) for TCS-associated AMI. The identification of celecoxib as a potential agent to mitigate TCS-induced cardiac injury further highlights the translational potential of our research. This work contributes to the growing body of evidence linking environmental toxicant exposure to cardiovascular disease and provides a foundation for future studies aimed at mitigating TCS-associated health risks.

## Figures and Tables

**Table 1:** Binding Energies of Ligands and Receptors.

Protein	PDB/Uniprot ID	Center (X, Y, Z)	Size (X × Y × Z)
*BCL6*	7OKL	26, −15, 18	50 × 48 × 62
*CEBPD*	P49716	10, 10, −11	115 × 79 × 133
*JDP2*	Q8WYK2	5, 13, −6	93 × 80 × 103
*JUN*	5T01	−25, 8, 17	41 × 41 × 46
*PTGS2*	5F1A	31, 37, 242	24 × 30 × 24
*S100A8*/9	8SJB	3, 22, 18	69 × 62 × 59
*THBD*	P07204	218, 215, 157	51 × 48 × 50

## Data Availability

The data presented in this study are available on request from the corresponding author. The data are not publicly available due to the need for further research utilization and subsequent project development. Gene expression datasets (GSE48060, GSE60993, GSE61144, GSE66360, GSE97320, GSE61145) can be accessed from the NCBI GEO repository (https://www.ncbi.nlm.nih.gov/geo/, accessed on 12 July 2025). The 3D structure of TCS (ligand) is available from the PubChem database (https://pubchem.ncbi.nlm.nih.gov/, accessed on 5 July 2025), and protein structures are available from the PDB database (https://www.rcsb.org/, accessed on 10 August 2025) or UniProt database (https://www.uniprot.org/, accessed on 10 August 2025). Data analysis was performed using R software 4.3.1, and the schematic figures in this manuscript were created with BioRender (https://BioRender.com/, accessed on 25 August 2025). The Academic Publication License (Agreement number: BU29CL3SZS) has been obtained, and the official citation is: Created in BioRender. Qi, Z. (2026). https://BioRender.com/s7qqurf. Custom code and processed data used in this study are available from the corresponding author upon reasonable request.

## References

[1] Weatherly L.M., Gosse J.A. (2017). Triclosan exposure, transformation, and human health effects. J. Toxicol. Environ. Health B Crit. Rev..

[2] Olaniyan L.W., Mkwetshana N., Okoh A.I. (2016). Triclosan in water, implications for human and environmental health. Springerplus.

[3] Marques A.C., Mariana M., Cairrao E. (2022). Triclosan and Its Consequences on the Reproductive, Cardiovascular and Thyroid Levels. Int. J. Mol. Sci..

[4] Zachariah J.P., Jone P.N., Agbaje A.O., Ryan H.H., Trasande L., Perng W., Farzan S.F. (2024). Environmental Exposures and Pediatric Cardiology: A Scientific Statement from the American Heart Association. Circulation.

[5] Dai C., Sun J., Yang G., Zhang C., Zhang Y., Song Q., Liu X., Duan X., Yang H., Li A. (2025). The total xanthones from Gentianella acuta alleviate acute myocardial infarction by targeting BRD4-mediated cardiomyocyte pyroptosis and inflammation. Phytomedicine.

[6] Khan M.I., Pathania S., Al-Rabia M.W., Ethayathulla A.S., Khan M.I., Allemailem K.S., Azam M., Hariprasad G., Imran M.A. (2024). MolDy: Molecular dynamics simulation made easy. Bioinformatics.

[7] Libby P., Buring J.E., Badimon L., Hansson G.K., Deanfield J., Bittencourt M.S., Tokgözoğlu L., Lewis E.F. (2019). Atherosclerosis. Nat. Rev. Dis. Primers.

[8] Diao W., Yan J., Wang X., Qian Q., Wang H. (2023). Mechanisms regarding cardiac toxicity triggered by up-regulation of miR-144 in larval zebrafish upon exposure to triclosan. J. Hazard. Mater..

[9] D’Antonio G., Di Fazio N., Pellegrini L., Ghamlouch A., Del Duca F., La Russa R., Frati P., Maiese A., Volonnino G. (2025). Immunohistochemical Assessment of Acute Myocardial Infarction: A Systematic Review. Int. J. Mol. Sci..

[10] Bai C., Wu L., Li R., Cao Y., He S., Bo X. (2025). Machine Learning-Enabled Drug-Induced Toxicity Prediction. Adv. Sci..

[11] Fang Y., Wang J., Liu C., Yin L., Li Y., Ji C., Zhou M., Zhou M., Hu Q. (2025). Targeting P2Y14R alleviates platelet-induced NET formation and venous thrombosis through PKA/AKAP13/RhoA axis. Eur. Heart J..

[12] Calafat A.M., Ye X., Wong L.Y., Reidy J.A., Needham L.L. (2008). Urinary concentrations of triclosan in the U.S. population: 2003–2004. Environ. Health Perspect..

[13] Saletti M., Maramai S., Reale A., Paolino M., Brogi S., Di Capua A., Cappelli A., Giorgi G., D’Avino D., Rossi A. (2022). Novel analgesic/anti-inflammatory agents: 1,5-Diarylpyrrole nitrooxyethyl sulfides and related compounds as *cyclooxygenase-2* inhibitors containing a nitric oxide donor moiety endowed with vasorelaxant properties. Eur. J. Med. Chem..

[14] Zhu L., Shao Y., Xiao H., Santiago-Schübel B., Meyer-Alert H., Schiwy S., Yin D., Hollert H., Küppers S. (2018). Electrochemical simulation of triclosan metabolism and toxicological evaluation. Sci. Total Environ..

[15] Liu Y., Wang C., Fu Z., Bai Y., Zheng G., Wu F. (2025). Common antimicrobials disrupt early zebrafish development through immune-cardiac signaling. Environ. Sci. Ecotechnol.

[16] Zhu M., Li Y., Xu Q., Wang W., Liu Y., Liu Y. (2025). Acute Myocardial Infarction: Molecular Pathogenesis, Diagnosis, and Clinical Management. MedComm.

[17] Villa E., Saso L., Chichiarelli S., Rojas-Solé C., Pinilla-González V., Prieto J.C., Gajardo A.I.J., Aguayo R., Rodrigo R. (2025). Antioxidant Cardioprotection in Acute Myocardial Infarction: From Mechanisms to Therapeutic Strategies. Front. Biosci..

[18] Cozac D.A., Halațiu V.B., Scridon A. (2025). The alarmin tandem: Unraveling the complex effect of *S100A8*/A9—From atherosclerosis to cardiac arrhythmias. Front. Immunol..

[19] Li D., Zhou J., Yang B., Yu Y. (2019). microRNA-340-5p inhibits hypoxia/reoxygenation-induced apoptosis and oxidative stress in cardiomyocytes by regulating the Act1/NF-κB pathway. J. Cell Biochem..

[20] Katashima T., Naruko T., Terasaki F., Fujita M., Otsuka K., Murakami S., Sato A., Hiroe M., Ikura Y., Ueda M. (2010). Enhanced expression of the *S100A8*/A9 complex in acute myocardial infarction patients. Circ. J..

[21] Narasimha A., Watanabe J., Lin J.A., Hama S., Langenbach R., Navab M., Fogelman A.M., Reddy S.T. (2007). A novel anti-atherogenic role for *COX-2*—Potential mechanism for the cardiovascular side effects of *COX-2* inhibitors. Prostaglandins Other Lipid Mediat..

[22] Cipollone F., Fazia M.L. (2006). *COX-2* and atherosclerosis. J. Cardiovasc. Pharmacol..

[23] Guillén J., Bernabeu A., Shapiro S., Villalaín J. (2004). Location and orientation of Triclosan in phospholipid model membranes. Eur. Biophys. J..

[24] Belosludtsev K.N., Belosludtseva N.V., Tenkov K.S., Penkov N.V., Agafonov A.V., Pavlik L.L., Yashin V.A., Samartsev V.N., Dubinin M.V. (2018). Study of the mechanism of permeabilization of lecithin liposomes and rat liver mitochondria by the antimicrobial drug triclosan. Biochim. Biophys. Acta Biomembr..

[25] Stachurski P., Kurach Ł., Khalavka M., Ptasiewicz M., Świątkowski W., Żelazowska R., Magryś A. (2025). Comprehensive assessment of triclosan-induced toxicity: Impacts on zebrafish development, mammalian cell viability and microbial activity. Adv. Med. Sci..

